# Ino80 is essential for proximal-distal axis asymmetry in part by regulating Bmp4 expression

**DOI:** 10.1186/s12915-016-0238-5

**Published:** 2016-03-14

**Authors:** Zhijun Qiu, Zeinab Elsayed, Veronica Peterkin, Suehyb Alkatib, Dorothy Bennett, Joseph W. Landry

**Affiliations:** Department of Human and Molecular Genetics, Virginia Institute of Molecular Medicine, Massey Cancer Center, Virginia Commonwealth University School of Medicine, Richmond, VA 23298 USA

**Keywords:** Ino80, Chromatin remodeling, Bmp4, DVE, PD Axis, Gastrulation, Embryonic ectoderm

## Abstract

**Background:**

Understanding how embryos specify asymmetric axes is a major focus of biology. While much has been done to discover signaling pathways and transcription factors important for axis specification, comparatively little is known about how epigenetic regulators are involved. Epigenetic regulators operate downstream of signaling pathways and transcription factors to promote nuclear processes, most prominently transcription. To discover novel functions for these complexes in axis establishment during early embryonic development, we characterized phenotypes of a mouse knockout (KO) allele of the chromatin remodeling Ino80 ATPase.

**Results:**

*Ino80* KO embryos implant, but fail to develop beyond the egg cylinder stage. *Ino80* KO embryonic stem cells (ESCs) are viable and maintain alkaline phosphatase activity, which is suggestive of pluripotency, but they fail to fully differentiate as either embryoid bodies or teratomas. Gene expression analysis of *Ino80* KO early embryos by in situ hybridization and embryoid bodies by RT-PCR shows elevated *Bmp4* expression and reduced expression of distal visceral endoderm (DVE) markers *Cer1*, *Hex,* and *Lefty1*. In culture, *Bmp4* maintains stem cell pluripotency and when overexpressed is a known negative regulator of DVE differentiation in the early embryo. Consistent with the early embryo, we observed upregulated *Bmp4* expression and down-regulated *Cer1*, *Hex,* and *Lefty1* expression when *Ino80* KO ESCs are differentiated in a monolayer. Molecular studies in these same cells demonstrate that Ino80 bound to the *Bmp4* promoter regulates its chromatin structure, which correlates with enhanced SP1 binding. These results in combination suggest that Ino80 directly regulates the chromatin structure of the *Bmp4* promoter with consequences to gene expression.

**Conclusions:**

In contrast to *Ino80* KO differentiated cells, our experiments show that undifferentiated *Ino80* KO ESCs are viable, but fail to differentiate in culture and in the early embryo. *Ino80* KO ESCs and the early embryo up-regulate *Bmp4* expression and down-regulate the expression of DVE markers *Cer1*, *Hex* and *Lefty1*. Based on this data, we propose a model where the Ino80 chromatin remodeling complex represses *Bmp4* expression in the early embryo, thus promoting DVE differentiation and successful proximal-distal axis establishment. These results are significant because they show that epigenetic regulators have specific roles in establishing embryonic axes. By further characterizing these complexes, we will deepen our understanding of how the mammalian embryo is patterned by epigenetic regulators.

**Electronic supplementary material:**

The online version of this article (doi:10.1186/s12915-016-0238-5) contains supplementary material, which is available to authorized users.

## Background

Mammalian embryonic development is best understood using the mouse model [1]. Fertilized eggs develop into a blastocyst by embryonic day 3.5 (E3.5). At this stage, the blastocyst is composed of both an inner cell mass (ICM), which is fated to become the embryo, and an extraembryonic trophectoderm (TE). The blastocyst implants into the uterus at E4.5, and during this time the embryo forms the epiblast (EPI) and primitive endoderm (PrE). During implantation the EPI rapidly proliferates and expands to form an egg cylinder, which is composed of both embryonic ectoderm (EmE) and extraembryonic ectoderm (ExE) covered by visceral endoderm (VE). During egg cylinder expansion, the distal tip of the embryo differentiates into the distal visceral endoderm (DVE), establishing a proximal-distal (P-D) axis. During the transition from E5.5 to E6.0, the DVE migrates up the anterior portion of the embryo to create the anterior visceral endoderm (AVE) where it has organizer activity through secreting several inhibitors of the Tgfb and Wnt family ligands. The secretion of these inhibitors constrains Tgfb and Wnt signaling activity to the posterior portion of the embryo, which promotes the differentiation of the primitive streak and the anterior-posterior (A-P) axis.

Early mammalian development, and the establishment of asymmetric axes (P-D and A-P axes), require coordinated gene expression [2]. Gene expression is regulated by both transcription factors and epigenetic regulators. Both operate within chromatin, which is composed of nucleosomes at its fundamental level [3, 4]. Essential epigenetic regulators in eukaryotes are chromatin remodeling complexes. Chromatin remodeling complexes are usually multi-subunit enzymes that slide or evict nucleosomes, or exchange its histone subunits. These activities change chromatin structure by altering the position, occupancy or composition of nucleosomes [5]. In turn, changes in chromatin structure regulate access to the underlying DNA, that in turn influences nuclear processes like transcription.

Chromatin remodeling complexes are classified into the SWI/SNF, ISWI, CHD or INO80 families based upon the sequence homology of their ATPase subunit [5]. In mammals, the INO80 family is composed of the Srcap, p400, and Ino80 remodeling complexes. These complexes are large, 12-15 subunit, complexes that are unique among chromatin remodeling complexes because they catalyze histone exchange reactions [6]. In addition to histone exchange, Ino80 has significant nucleosome sliding activity, suggesting that it can either alter nucleosome position or occupancy, or change nucleosome composition in vivo. Presumably through these activities, Ino80 regulates a variety of nuclear processes which include transcription, DNA repair, DNA replication, and telomere structure [6].

While much has been done to characterize the nuclear functions of Ino80, little has been done to determine its functions in metazoan development. In plants, INO80 is essential for flowering and reproductive organ development, possibly through functions in homologous recombination and regulated transcription [7, 8]. In flies, INO80 mutants are late embryonic lethal, deregulate Hox gene expression, and manifest homeotic transformations [9]. In addition to Hox genes, INO80 regulates ecdysone response genes, which are essential for pupal development and molting [10]. Localization and nucleosome mapping studies in insect cells show that INO80 is widely distributed throughout the genome and remodels nucleosomes onto energetically unfavorable DNA sequences [11]. Similar to studies in plants and flies, mammalian Ino80 is also essential for development. Ex vivo studies with Ino80 knockout (KO) or shRNA knockdown (KD) pre-implantation embryos show that Ino80 maintains stem self-renewal by promoting the expression of pluripotency factors like Oct4 and stabilizing DNA replication forks [12, 13]. In utero, *Ino80* KO embryos implant into the uterus but fail to develop to mid-gestation, possibly due to roles for Ino80 in regulating telomere structure or DNA damage repair [14]. Because Ino80 is a well-documented regulator of gene expression, developmental defects could also result from abnormal gene expression [13].

Building on these earlier studies, we show that *Ino80* KO embryos fail to specify a DVE and a P-D axis in utero. Coincident with defects in the DVE, *Ino80* KO embryos aberrantly express *Bmp4* in the EmE, a known repressor of DVE specification [15]. Molecular studies using differentiating ESC models show that Ino80 is specifically localized to the *Bmp4* promoter, remodels its chromatin structure, and regulates the binding of transcription activators to its DNA sequence. These results in combination suggest that Ino80 directly represses *Bmp4* expression in the EmE through its chromatin remodeling activity to promote DVE specification and P-D axis establishment.

## Results

### *Ino80* KO embryos fail to develop beyond the egg cylinder stage

To discover the functions of Ino80 in mammalian development, we created a conditional KO allele in mice using Cre-loxp technology [16]. With this strategy, essential exons are flanked by loxp sites that can be excised by tissue-specific Cre recombinase expression. The targeted Floxed-Neo allele (Neo) is designed to delete exons 2-4 of the *Ino80* gene with Cre excision, deleting the initiating ATG (Additional file 1a). To create both the *Ino80* Floxed allele (F) and the *Ino80* deletion allele (KO), we crossed mice carrying our targeted allele to mice constitutively expressing the Flp or Cre recombinases. Successful recombination and germ line transmission of the Floxed and KO alleles were identified by Southern blotting and PCR-based genotyping strategies (Additional file 1b-d).

To determine if our targeted allele resulted in a loss of Ino80 protein, we crossed a Tet inducible Cre expression system (M2-rtTA and TetO-Cre alleles) into our *Ino80* Floxed mice [17, 18]. Homozygous *Ino80* Floxed (F/F) mouse embryonic fibroblasts (MEFs), with or without TetO-Cre, were isolated from mid-gestation E12.5 embryos. As expected, the addition of doxycycline to TetO-Cre, *Ino80* F/F fibroblast cultures resulted in the conversion of the *Ino80* Floxed alleles to *Ino80* KO alleles in two days (Additional file 2a). Over this same time course, Western blotting demonstrated a complete depletion of the Ino80 protein by day 6 (Additional file 2b). Coincident with the depletion of the Ino80 protein, we observed a loss of the exons 1-7 *Ino80* transcript and the appearance of two smaller *Ino80* transcripts (KO-1 and KO-2) by RT-PCR (Additional file 2c, d). Cloning and sequencing these smaller PCR products identified aberrant exon 1-5 and exon 1-6 splice events for the *Ino80* transcript, with the deletion of exons 2-4 by Cre-mediated recombination (Additional file 2d). In silico translation from the first ATG of each aberrant splice product results in an out of frame transcript and a random protein product (Additional file 2e). These results demonstrate that our *Ino80* Floxed allele can be successfully excised by Cre recombinase, resulting in loss of the Ino80 protein.

**Fig. 1:**
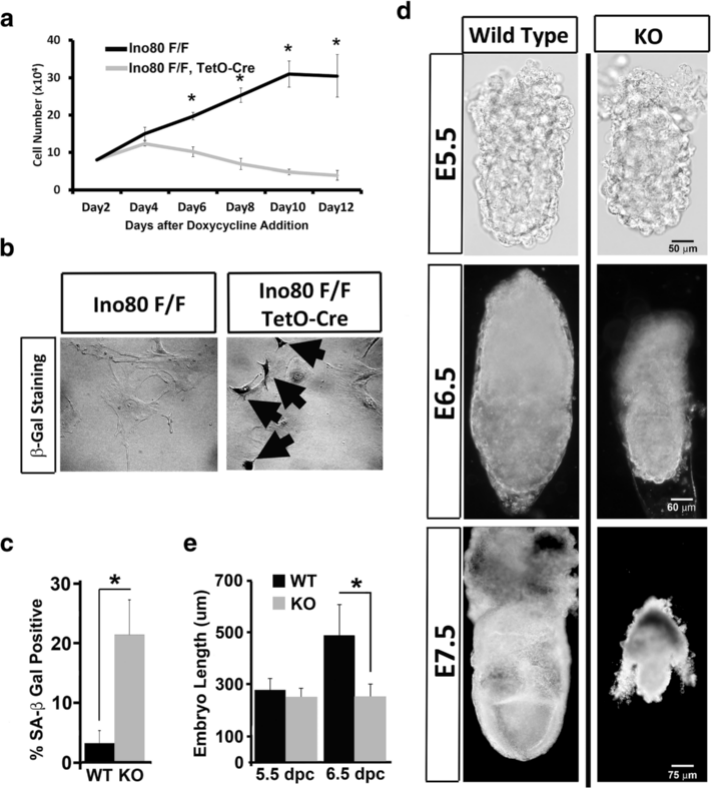
Ino80 is essential for embryonic fibroblast and early embryonic development. **a** Growth curve of conditional *Ino80* KO MEF induced by doxycycline treatment. 1.0 × 10^4^ cells of control (*Ino80* F/F) or *Ino80* KO (*Ino80* F/F, TetO-Cre) were seeded into media containing 10 μg/ml doxycycline. Numbers of trypan blue negative cells were counted every two days over a 12-day period (*N* = 3 independent measurements per group, representative of three biological replicates; * = *t* test *p* ≤ 0.05). **b** Day-12 control and *Ino80* KO cells from panel a were stained for endogenous β-galactosidase activity at pH 6.0 to measure cellular senescence [19]. *Black arrows* designate β-galactosidase positive cells. **c** Quantification of percentage β-galactosidase positive cells for day-12 control and *Ino80* KO cells from panel (**b**) (*N* = 5 independent measurements per group, representative of three biological replicates; * = *t* test: *p* ≤ 0.05). **d** Whole mount analysis of E5.5-, E6.5-, and E7.5-day wild-type (+/+) and homozygous *Ino80* KO (*Ino80* KO/*Ino80* KO) embryos harvested from +/*Ino80* KO intercross breeding. **e** Length of E5.5- and E6.5-day wild-type and *Ino80* KO embryos were measured in whole mount using a calibrated microscope (*N* > 7 embryos per group; * = *t* test *p* ≤ 0.05). *KO* knockout, *MEF* mouse embryonic fibroblasts, *E* embryonic day

A previous publication showed that MEFs depleted of Ino80 arrest the cell cycle and senesce [14]. To determine if depletion of Ino80 using our *Ino80* Floxed allele resulted in similar phenotypes, we counted the number of viable fibroblasts over 12 days post-Cre expression by doxycycline exposure. As described previously [14], we observed that after doxycycline exposure *Ino80* KO MEFs stopped proliferating and senesced as identified by an increased number of cells positive for endogenous b-Gal activity, an indicator of senescence [19] (Fig. 1a-c). Reproducing previous phenotypes of *Ino80* KO MEFs supports the conclusion that our *Ino80* KO allele is a null allele.

Phenotypes for *Ino80* KO embryos are largely uncharacterized. To identify the functions of Ino80 in mammalian development, we intercrossed mice heterozygous for the *Ino80* KO allele. Genotyping litters of mice from this intercross identified no homozygous *Ino80* KO mice, demonstrating that Ino80 is essential for mouse viability (Additional file 3). To stage the earliest point where Ino80 is required for development, we genotyped litters of embryos at mid-gestation (E13.5), gastrulation (E6.5), and peri-implantation (E4.5). Results from these experiments showed that *Ino80* KO homozygous embryos were absent at E13.5, but present at the expected Mendelian ratios at gastrulation (E6.5) and prior to implantation (E4.5) (Additional file 3). These results demonstrate that Ino80 is essential for mammalian development post-gastrulation. A gross analysis of *Ino80* KO embryos prior to (E5.5), during (E6.5), and post-gastrulation (E7.5) reveled that Ino80 is important for expansion of the egg cylinder during gastrulation (Fig. 1d). Quantitation of embryo length showed that the size of *Ino80* KO embryos at E6.5 is similar to that observed for *Ino80* KO embryos at E5.5 (Fig. 1e). Embryo reabsorption likely occurs after E7.5 because *Ino80* KO embryos after E7.5 begin to show a loss in structural integrity (data not shown).

### *Ino80* KO embryonic stem cells are viable but exhibit an unstable pluripotent state

**Fig. 2:**
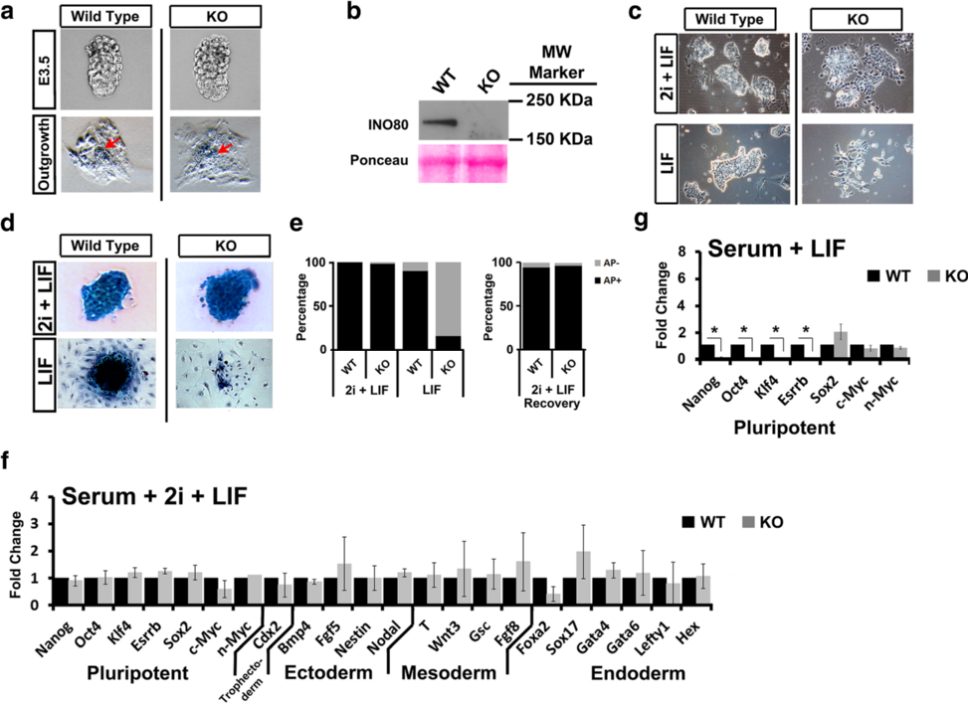
*Ino80* KO ESCs are viable, but differentiate when maintained in a metastable pluripotent state. **a** Microscopic analysis of wild-type and *Ino80* KO pre-implantation E3.5 blastocysts. Blastocysts were allowed to outgrow for seven days onto gelatinized plates in media containing serum + LIF. Wild-type and *Ino80* KO are at identical magnification. *Red arrows* designate ESC colony outgrowth. **b** Western analysis of Ino80 protein expression in wild-type and *Ino80* KO ESCs using a custom Ino80 antibody. Ponceau S was used as a loading control. **c** Microscope analysis of wild-type and *Ino80* KO ESCs grown in media containing serum + 2i + LIF or serum + LIF. Wild-type and *Ino80* KO are at identical magnification. **d** Alkaline phosphatase (AP) activity in wild-type and *Ino80* KO ESCs after plating at clonal density and maintained in media containing serum + 2i + LIF or serum + LIF. Wild-type and *Ino80* KO are at identical magnification. **e** Percent AP positive colonies quantified from panel d and after *Ino80* KO ESCs passaged in serum + LIF were recovered in serum + 2i + LIF. At least 50 colonies were quantified per group. **f** Quantitative RT-PCR analysis of gene expression in wild-type and *Ino80* KO ESCs maintained in serum + 2i + LIF. (N = 3 biological replicates) **g** Quantitative RT-PCR analysis of gene expression in wild-type and *Ino80* KO ESCs maintained in serum + LIF. (*N* = 3 biological replicates; * = *t* test *p* ≤ 0.05). *KO* knockout, *ESC* embryonic stem cells, *E* embryonic day, *LIF* leukemia inhibitory factor,

To characterize roles for Ino80 in the early embryo, we isolated *Ino80* KO embryonic stem cells (ESCs) from the ICM of pre-implantation E3.5 *Ino80* KO embryos. Collection and genotyping pre-implantation E3.5-day blastocysts from *Ino80* KO heterozygous intercrosses showed that *Ino80* KO homozygous blastocysts look morphologically similar to wild-type littermates by gross inspection (Fig. 2a). These same isolated blastocysts successfully attached and outgrew onto gelatinized plates in media containing serum and leukemia inhibitory factor (LIF). After seven days of outgrowth, we observed a similar expansion of trophoblasts and the ICM between wild-type and *Ino80* KO blastocysts (Fig. 2a). From these outgrowths, we successfully cloned *Ino80* KO ESCs under culture conditions that maintain ground state pluripotency (media containing serum + 2i + LIF) [20, 21]. Western blotting showed that the *Ino80* KO ESCs do not have the Ino80 protein, further confirming that our *Ino80* KO allele is a null allele (Fig. 2b). Unlike *Ino80* KO MEFs, *Ino80* KO ESCs proliferate and are not apoptotic when maintained in culture under conditions that promote ground state pluripotency (Additional file 4a, b). A microscopic analysis shows that *Ino80* KO ESCs did not form prototypical undifferentiated colonies and exhibit slight cell scattering when maintained at ground state pluripotency (Fig. 2c). Cell scattering is more pronounced when the ESCs were passaged in a media formulation that maintains a metastable pluripotent state (serum + LIF) (Fig. 2c). The percentage of colonies that maintain alkaline phosphatase (AP) staining was equivalent between wild-type and *Ino80* KO ESCs when maintained at ground state pluripotency (Fig. 2d, e). However, *Ino80* KO ESCs did not form robust AP positive colonies if the colony formation assay was repeated when cells were maintained in a metastable state (Fig. 2d, e). The ability to form AP positive colonies was recovered if cells maintained in the metastable state are returned to ground state conditions (Fig. 2e). These results demonstrate that *Ino80* KO ESCs are viable, AP positive, and morphologically show evidence of slight cell scattering when compared to wild-type controls when maintained at ground state pluripotency. However, *Ino80* KO ESCs lose AP staining and exhibit cell scattering when grown under conditions that maintain a metastable pluripotent state.

To further characterize phenotypes of *Ino80* KO ESCs grown under differing culture conditions, we measured transcript levels of several markers of pluripotency, stem cell differentiation, and lineage commitment. When maintained at ground state pluripotency, *Ino80* KO ESCs have equivalent expression of pluripotency markers *Nanog*, *Oct4*, *Klf4*, *Sox2,* and *Essrb*. Markers of ectoderm, mesoderm, and endoderm lineages were also approximately equivalent between wild-type and *Ino80* KO ESCs (Fig. 2f). In contrast, we observed reduced pluripotency marker expression (*Nanog*, *Oct4*, *Klf4,* and *Essrb*) when *Ino80* KO ESCs were maintained in the metastable state for five days (Fig. 2g) [13]. These results are consistent with the metastable pluripotent state, but not ground state pluripotency requiring Ino80.

### *Ino80* KO ESCs fail to differentiate using models of mammalian development

**Fig. 3:**
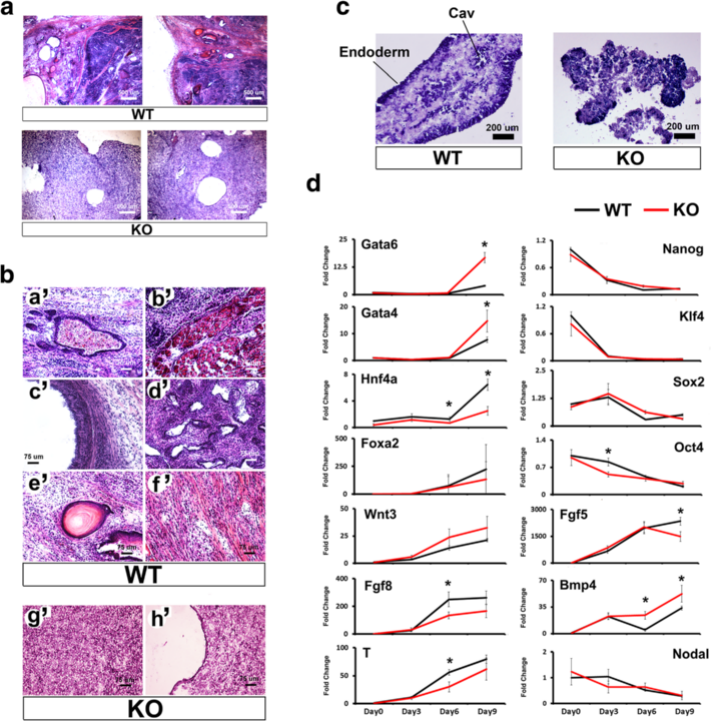
Ino80 is essential for cell and tissue differentiation. **a** Low-magnification analysis of wild-type and *Ino80* KO ESC differentiation as teratomas in NOD/SCID mice. Scale bar = 500 μm. **b** High-magnification analysis of teratomas derived from wild-type ESCs showed evidence of (*a’*) blood islands, (*b’*) striated muscle, (*c’*) ciliated endoderm, (*d’*) neural rosettes, (*e’*) keratin pearls, and (*f’*) smooth muscle fields when sectioned and stained with hematoxylin and eosin (H + E). Teratomas formed from *Ino80* KO ESCs are composed of (*g’*) undifferentiated tissue surrounded by a (*h’*) thin epithelial cell layer. Scale bar = 75 μm. **c** Analysis of 9-day embryoid bodies derived from wild-type and *Ino80* KO ESCs. Frozen sections of embryoid bodies derived from wild-type ESCs stained with H + E showed evidence of dark staining endoderm (Endoderm) and evidence of internal cavitation (Cav) as previously described [24]. Embryoid bodies from *Ino80* KO ESCs show evidence of disorganized endoderm and other differentiated cell types. Scale bar = 200 μm. **d** Gene expression analysis of embryoid bodies derived from either wild-type or *Ino80* KO ESCs differentiated over nine days. Quantitative RT-PCR was used to measure the expression of a variety of differentiation markers, including Gata*6*, *Gata4*, *Foxa2, Hnf4a*, *Wnt3*, *Fgf8*, *T*, *Fgf5*, *Bmp4*, and *Nodal,* and pluripotency markers, including *Nanog*, *Klf4*, *Sox2* and *Oct4*. (Representative of *N* = 3 biological replicates; * = *t* test *p* ≤ 0.05). *KO* knockout, *ESC* embryonic stem cell

To further characterize roles for Ino80 during mammalian development, we differentiated *Ino80* KO ESCs using the teratoma model [22]. To create teratomas, both wild-type and *Ino80* KO ESCs maintained at ground state were introduced into opposing flanks of NOD/SCID mice. Teratomas were allowed to grow until control tumors were approximately 1 cm in diameter. From five inoculations, we harvested four wild-type tumors and two *Ino80* KO tumors. A histological analysis showed that control tumors formed a wide range of differentiated tissues from each of the three germ lines. Differentiated tissues include blood islands, keratin pearls, and neural rosettes from the ectoderm lineage, both striated and smooth muscle from mesoderm, and ciliated endoderm from the endoderm lineage (Fig. 3a, b). In contrast to the well-differentiated tissues observed in wild-type teratomas, we observed that *Ino80* KO teratomas are composed of undifferentiated mesenchyme with a thin layer of epithelial cells on the outer periphery (Fig. 3a, b).

In addition to forming teratomas, we utilized the embryoid body ESC differentiation model [23]. To create embryoid bodies, ESCs maintained at ground state were dispersed in clumps and aggregated in serum containing media without 2i + LIF. ESC aggregates were then harvested at three-day intervals over the course of nine days. Histological analysis of day-9 wild-type embryoid bodies provided evidence of differentiated cell types including a well-defined endoderm, and an organized mesenchyme with evidence of cavitation as previously described [24] (Fig. 3c). In contrast, embryoid bodies derived from *Ino80* KO ESCs were not well organized and lacked a continuous endoderm (Fig. 3c). We subsequently used RT-PCR to measure markers of pluripotency and differentiated tissues in the embryoid bodies. From these experiments, we observed that pluripotency marker repression during embryoid body differentiation is largely Ino80-independent (Fig. 3d). In contrast to pluripotency markers, we observed Ino80-dependent expression for several differentiation markers during embryoid body differentiation. *Ino80* KO embryoid bodies showed enhanced expression of endoderm markers *Gata6* and *Gata4* [25], and the stem cell maintenance factor *Bmp4* (Fig. 3d) [26]. The expression of these markers could be linked since *Bmp4* positively regulates *Gata4* and *Gata6* expression [27]. In contrast to these changes, *Ino80* KO embryoid bodies showed reduced expression of the ectoderm marker *Fgf5* [28], the endoderm marker *Hnf4a* [29], and mesoderm markers *Fgf8* [30] and *T* [31] (Fig. 3d). Because the expression of these markers is essential for embryonic development, Ino80-dependece of these same genes could contribute to a post-implantation lethal phenotype.

### *Ino80* is expressed in the embryonic and extraembryonic ectoderm of post-implantation embryos

**Fig. 4:**
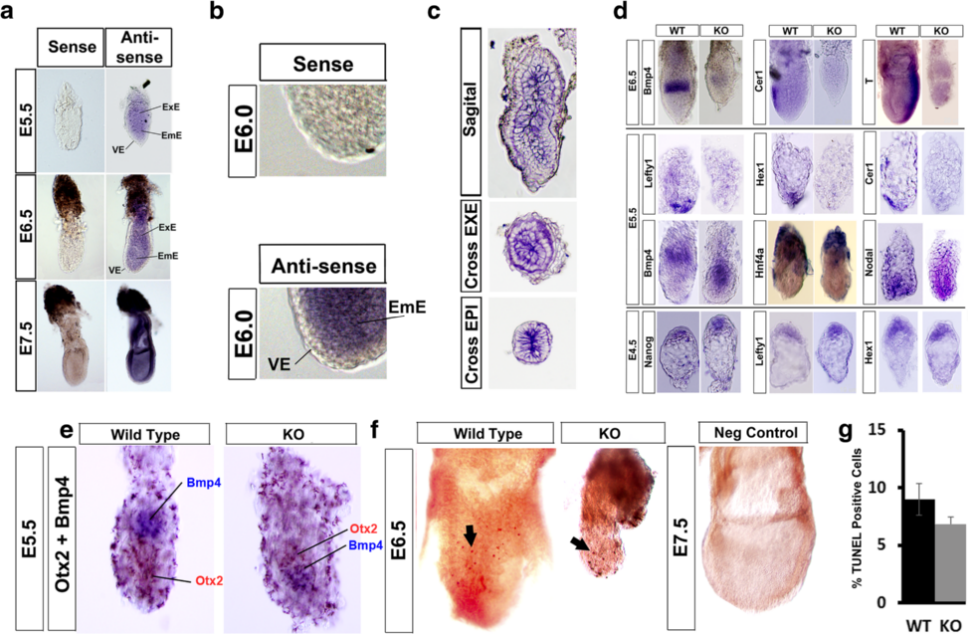
*Ino80* is widely expressed in embryonic tissues and is essential for establishing the proximal-distal axis of the post-implantation embryo. **a** Whole mount RNA in situ hybridization (ISH) of E5.5-, E6.5-, and E7.5-day embryos using either sense or antisense *Ino80* riboprobes. All embryos were photographed at identical magnification. Extraembryonic ectoderm (ExE), embryonic ectoderm (EmE) and visceral endoderm (VE) are designated. **b** Increased magnification showing reduced antisense *Ino80* riboprobe staining in the visceral endoderm (VE) in E6.0-day embryos. Embryos were photographed at identical magnification. **c** Frozen sections of E6.5-day embryos stained whole mount with antisense *Ino80* riboprobe. Sagittal and cross sections through the ExE and EPI were obtained for representative embryos. **d** Whole mount RNA in situ hybridization of E6.5-, E5.5-, and E4.5-day embryos was performed with antisense riboprobes for markers of tissue differentiation. Markers used include the ExE marker (*Bmp4)*, EmE marker (*Nodal)*, mesoderm marker (*T)*, EPI marker (*Nanog)*, VE marker (*Hnf4a*), and PrE/DVE/AVE markers (*Cer1*, *Lefty1,* and *Hex1)*. Embryos were photographed at identical magnification. **e** Whole mount ISH of E5.5-day embryos was performed with antisense probes to the ExE marker *Bmp4* (*purple color*) and the EmE marker *Otx2* (*magenta color*). Embryos were photographed at identical magnification. **f** Whole mount analysis of TUNEL positive cells in wild type and *Ino80* KO E6.5-day embryos. A representative negative control (- TdT enzyme) is shown. Representative TUNEL positive cells are shown by *black arrows*. **g** Quantitation of apoptosis as percentage of TUNEL positive cells from panel f (see methods section). *N* = 2 wild type (WT) or *Ino80* KO embryos. *E* embryonic day, *EPI* epiblast, *PrE* primitive endoderm, *DVE* distal visceral endoderm, AVE anterior visceral endoderm

In a first step towards understanding why *Ino80* KO embryos fail to develop beyond E6.5, we determined where *Ino80* is expressed during embryonic development and in the adult. Towards this end, we performed in situ RNA hybridization (ISH) using both sense and antisense probes to the *Ino80* transcript in E5.5- to E7.5-day embryos. Whole mount ISH of E5.5 to E6.5 with sense and antisense *Ino80* probes showed widespread staining of embryos with the antisense probe, but not the sense probe (Fig. 4a). The most intense staining with the antisense probe was observed in both the EmE and ExE, and comparatively less staining was observed in the VE (Fig. 4a, b). The EmE contributes to all cell types of the embryo proper, whereas the ExE contributes to the placenta and umbilical cord [1]. To better visualize *Ino80* ISH, we sectioned E6.5-day embryos either sagittal or in cross section. Inspection of these sections showed that staining with the antisense *Ino80* probe is highest in the ExE and EmE, with less staining in the VE (Fig. 4c). Consistent with widespread expression in the early embryo, Northern blotting for *Ino80* transcripts showed that it is expressed in all adult tissues analyzed (Additional file 5). The combination of these results supports the conclusion that *Ino80* is widely expressed in many tissues of post-implantation embryo and the adult.

### *Ino80* KO embryos have a defective distal visceral endoderm and fail to gastrulate

To better understand why *Ino80* KO embryos fail to develop, we used whole mount ISH to monitor marker expression for several essential cell types of the gastrulating embryo. We focused our ISH on markers previously analyzed from our studies of *Ino80* KO embryoid bodies (Fig. 3d). In the early embryo, gastrulation occurs at E6.5 when the posterior of the embryo differentiates into mesoderm and definitive endoderm [1]. Initial ISH experiments documented a lack of mesoderm marker *T* expression in *Ino80* KO embryos, suggesting that they do not differentiate mesoderm and likely do not gastrulate (Fig. 4d). Gastrulation requires the specification of the AVE and is the first organizer of the mammalian embryo. The AVE secretes inhibitors to Tgfb and Wnt ligands including Cer, and its expression at E6.5 is a marker of the AVE [32]. At E6.5 *Cer1* is not expressed in the *Ino80* KO embryo, suggesting that Ino80 is required for AVE establishment (Fig. 4d). In addition to the AVE, signaling molecules that originate from the ExE are essential for AVE establishment and gastrulation [33]. One such molecule is the Tgfb superfamily member Bmp4 [34]. ISH for *Bmp4* transcripts showed that it is weakly expressed in the ExE in *Ino80* KO E6.5 embryos (Fig. 4d). The results from these analyses demonstrate that *Ino80* KO embryos do not gastrulate and have defects in specifying both the AVE and ExE.

To determine if defects in gastrulation result from defects at earlier stages of development, we measured tissue differentiation in *Ino80* KO E5.5 embryos. From these experiments, we observed that *Bmp4*, which is normally expressed in the ExE [34], is abnormally expressed in the EmE in *Ino80* KO E5.5 embryos (Fig. 4d). To further confirm EmE *Bmp4* expression we performed a double ISH using *Bmp4* and the EmE marker *Otx2* [35]. From this experiment we show co-localization of *Bmp4* and *Otx2* expression in *Ino80* KO E5.5-day embryos (Fig. 4e). To further monitor for defects in the EmE, we next measured *Nodal* expression. At E5.5 *Nodal* is expressed in the EmE where it promotes proliferation of the epiblast and differentiates the DVE [36]. In *Ino80* KO E5.5 embryos, *Nodal* was normally expressed suggesting that the EmE is partially specified (Fig. 4d). Previous reports have shown that over expression of *Bmp4* in the early embryo inhibits DVE differentiation [15]. To determine if *Ino80* KO embryos have a defective DVE, we measured the expression of DVE markers *Cer1*, *Hex1,* and *Lefty1* [37]. None of these markers is expressed in E5.5 *Ino80* KO embryos, suggesting that *Ino80* KO embryos do not specify a DVE (Fig. 4d). Defects in DVE specification could be due to a general defect in the VE because we observed reduced expression of the extraembryonic VE marker *Hnf4a* in *Ino80* KO embryos (Fig. 4d) [29]. These results demonstrate that the DVE does not form in *Ino80* KO E5.5 embryos, which could result from expression of the inhibitory molecule Bmp4 in the EmE and general defects in the VE.

Both the VE and the DVE originate from the PrE, which is specified during implantation at E4.5 [38]. To determine if defects in the DVE result from defects in the PrE, we stained *Ino80* KO E4.5 embryos with the PrE markers, *Lefty1* and *Hex1*. From these experiments, we observed equivalent expression of *Lefty1* and *Hex1,* suggesting that the PrE of *Ino80* KO embryos are specified at E4.5 (Fig. 4c). In addition to the PrE, the E4.5 embryo has an EPI that is the precursor to the E5.5 EmE [38]. We next determined if the EPI is specified by staining for *Nanog,* a marker of the ICM and EPI. From these experiments we observed equivalent expression of *Nanog* in the EPI, suggesting that it is specified in *Ino80* KO embryos (Fig. 4d).

We next stained E6.5-day embryos using TUNEL to determine if defects in development are due to increased apoptosis with *Ino80* KO. From these experiments we observed no increase of TUNEL positive cells between wild-type and *Ino80* KO embryos, suggesting that the observed defects are not due to increased cell death (Fig. 4f, g) and likely are due to reduced cell proliferation.

In combination, these expression studies demonstrate that *Ino80* KO embryos do not gastrulate. Defects in mesoderm differentiation coincide with a lack of the AVE and its organizer activity, and are not due to increased cell death. The lack of an AVE at E6.5 is due to the inability of *Ino80* KO embryos to specify a DVE at E5.5. The lack of a DVE coincides with the abnormal expression of *Bmp4* in the EmE, a known negative regulator of DVE differentiation [15]. Defects in the DVE are not likely due to defects prior to E5.5 because both the PrE and EPI appear to be normal in E4.5 *Ino80* KO embryos.

### Ino80 represses *Bmp4* expression during embryonic stem cell differentiation

**Fig. 5:**
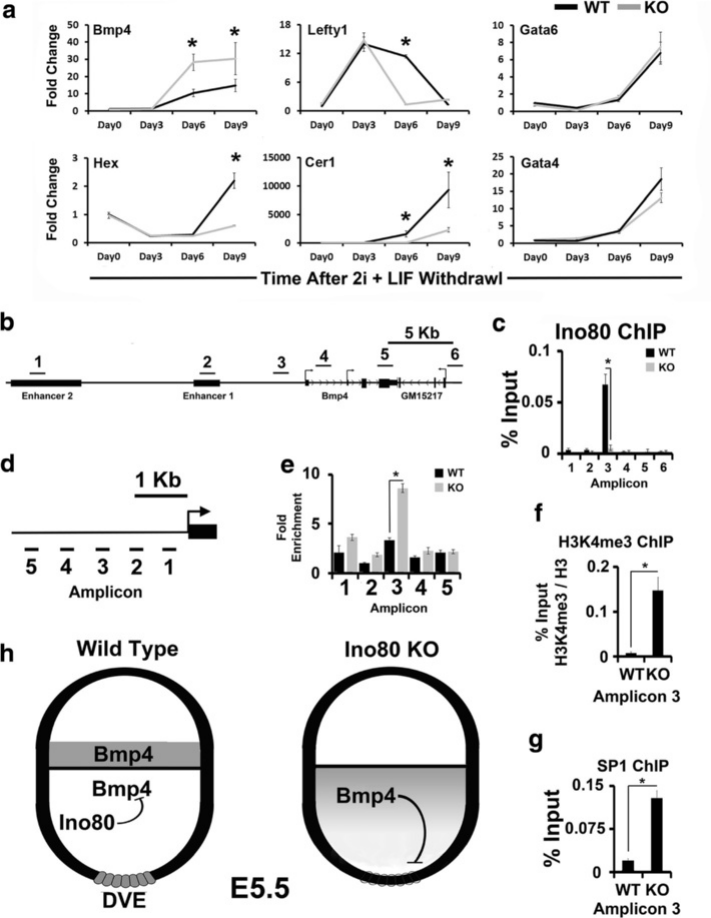
Ino80 localizes to *Bmp4* promoter and regulates its expression during embryonic stem cell differentiation. **a** Quantification of *Bmp4*, *Hex*, *Lefty1*, *Cer1*, *Gata6,* and *Gata4* expression by RT-qPCR during ESC differentiation in serum containing media without 2i + LIF in monolayer (Representative of *N* = 3 biological replicates; * = *t* test *p* ≤ 0.05). **b** Cartoon showing the *Bmp4* gene, location of upstream enhancers, and immediate downstream convergent gene *Gm15217* position of PCR amplicons used in chromatin immunoprecipitation (ChIP) experiments are shown as *black bars* labeled 1-6. **c** Localization of Ino80 to regulatory elements of *Bmp4* by chromatin immunoprecipitation (ChIP). ChIP was performed with wild-type and *Ino80* KO ESC after differentiation as a monolayer for six days in serum containing media lacking 2i + LIF. Position of PCR amplicons are shown in panel a (Representative of *N* = 3 biological replicates; * = *t* test *p* ≤ 0.05). **d** Cartoon showing the *Bmp4* promoter and the location of PCR amplicons used in FAIRE experiments are shown as *black bars* labeled 1-5. **e** Changes in chromatin structure ~1.5 Kb upstream of *Bmp4* in *Ino80* KO ESC. FAIRE was performed with wild-type and *Ino80* KO ESC after differentiation in serum containing media lacking 2i + LIF as a monolayer for six days. Position of PCR amplicons is shown in panel d (Representative of *N* = 3 biological replicates; * = *t* test *p* ≤ 0.05). **f** Increased H3K4me3 occupancy at *Bmp4* in *Ino80* KO ESC. ChIP was performed with wild-type and *Ino80* KO ESC after differentiation as a monolayer for six days in serum containing media lacking 2i + LIF. PCR amplicon 3 from panel b was used for ChIP (Representative of *N* = 3 biological replicates; * = *t* test *p* ≤ 0.05). **g** Increased SP1 occupancy at *Bmp4* in *Ino80* KO ESC. ChIP was performed with wild-type and *Ino80* KO ESC after differentiation in serum containing media lacking 2i + LIF as a monolayer for six days. PCR amplicon 3 from panel b was used for ChIP (Representative of *N* = 3 biological replicates; * = *t* test *p* ≤ 0.05). **h** Model for Ino80 function during proximal-distal axis establishment. In wild-type embryos, Ino80 in part functions in the EmE to repress *Bmp4* expression. *Bmp4* repression in the EmE promotes DVE differentiation. In *Ino80* KO embryos, *Bmp4* is abnormally expressed in the EmE, which inhibits DVE differentiation. A lack of a DVE prevents the AVE from forming, which subsequently prevents gastrulation. *ESC* embryonic stem cell, *LIF* leukemia inhibitory factor, *FAIRE* formaldehyde assisted isolation of regulatory elements, *KO* knockout, EmE embryonic ectoderm, *DVE* distal visceral endoderm, *AVE* anterior visceral endoderm

ISH experiments suggest that Ino80 represses *Bmp4* expression during embryoid body differentiation and in the EmE of early post-implantation embryos (Figs. 3d and 4d, e). These results suggest a model where Ino80 directly represses *Bmp4* expression in the EmE. To explore if Ino80 directly represses *Bmp4,* we used an in vitro model of early embryonic development. The differentiation of ESCs in monolayer by withdrawal of 2i + LIF in the presence of serum is a relevant model of early embryonic development. When ESCs are grown under these conditions, they differentiate into EPI lineages including EmE and mesoderm precursors [39]. Under these conditions, we observe elevated expression of *Bmp4* in *Ino80* KO ESCs (Fig. 5a). Elevated *Bmp4* expression coincides with repression of DVE markers *Cer1*, *Hex,* and *Lefty1*. Defects in DVE marker expression are not due to general defects in endoderm differentiation because we observed equivalent expression of endoderm markers *Gata4* and *Gata6* (Fig. 5a). In embryonic tissue lineages *Bmp4* is regulated by two upstream enhancers (Fig. 5b) [40]. Chromatin immunoprecipitation (ChIP) experiments showed that Ino80 is localized to the *Bmp4* promoter, but not the enhancers, when ESCs are differentiated for six days in serum containing media lacking 2i + LIF (Fig. 5c). Under these same conditions, we next used formaldehyde assisted isolation of regulatory elements (FAIRE) to detect differences in open chromatin at the *Bmp4* promoter in *Ino80* KO ESCs. From these studies, we detected increased open chromatin ~1.5 Kb upstream of the *Bmp4* transcription start site in *Ino80* KO ESCs (Fig. 5d, e). These changes in chromatin structure at ~1.5 Kb upstream of *Bmp4* correlated with increased H3K4me3 and SP1 occupancy (Fig. 5f, g). H3K4me3 is a well characterized mark of active promoters [41], and SP1 is a transcription factor known to activate *Bmp4* expression [42]. In combination, these experiments suggest that *Bmp4* is a direct target of Ino80 chromatin remodeling activity with consequences to SP1 binding and *Bmp4* expression. From these studies, we present a model where Ino80 normally represses *Bmp4* expression in the EmE. When Ino80 is deleted, *Bmp4* expression increases, DVE differentiation is repressed, and embryonic development is stopped (Fig. 5h).

## Discussion

In the last few decades it has been discovered that chromatin remodeling complexes are essential for many aspects of mammalian development [43, 44]. Several chromatin remodeling complexes are essential for pre-implantation development including SWI/SNF (*Brg1* KO) [45], NURD [46], and TIP60/p400 [47] complexes. In contrast, several other complexes including NURF (*Bptf* KO) [48] and CHD7 [49] have post-implantation phenotypes, suggesting that they regulate specific developmental pathways. Similarly, we showed that *Ino80* KO embryos implant, but fail to develop beyond the egg cylinder stage. These findings contrast with the cell essential functions for Ino80 in differentiated MEFs, where it regulates telomere structures and DNA replication [12, 14]. The viability of *Ino80* KO ESCs, but not MEFs, could be due to differences in telomere structure and DNA replication, which are known to occur between the cell types [50, 51].

To understand better how Ino80 regulates embryonic development, we derived *Ino80* KO ESCs from pre-implantation embryos. We maintained *Ino80* KO ESC under growth conditions that promote either ground state (serum + 2i + LIF) or metastable pluripotency (serum + LIF). Previous studies have shown that these culture conditions simulate growth conditions of the E3.5 blastocyst ICM (ground state) or E4.5 EPI and PrE (metastable state) [21]. *Ino80* KO ESCs remain undifferentiated at ground state pluripotency, but begin to show signs of differentiation in the metastable state, including loss of robust AP staining, and down-regulation of pluripotency markers *Nanog, Oct4, Klf4,* and *Esrrb*. These results support a model where Ino80 is not required to maintain the pluripotent E3.5 blastocyst ICM in utero, but rather has essential functions in regulating cellular differentiation of the E4.5 peri-implantation embryo. Our conclusions deviate slightly from previous studies which document that Ino80 is required to maintain ESC pluripotency and pre-implantation development [13]. Differences in methods used to achieve loss of function could explain these contrasting reports. The use of a siRNA KD approach could deplete maternal *Ino80* transcripts, thus revealing pre-implantation phenotypes [13]. Conversely, a genetic KO approach would leave maternal *Ino80* transcripts intact, mask any pre-implantation phenotypes, and allow the embryo to progress to post-implantation development (this study and [12, 14]).

In addition, the discrepancy could arise because Ino80 is required for *Oct4*, *Nanog,* and *Sox2* expression in the metastable state (serum + LIF) [13], but not the ground state (2i + LIF) [this study]. The ability of 2i + LIF to mask the self-renewal defects of *Ino80* KO ESC is not unprecedented as it has also been reported for *Mbd3* KO ESC [46, 52]. In the metastable state, FGF4/MAPK signaling upregulates pro differentiation transcription factors promoting differentiation [53, 54]. It is plausible that depletion of Ino80, and the accompanying reduction in *Oct4* expression [13], could synergize with aberrantly expressed pro differentiation factors to promote differentiation in the metastable state. Alternatively, increased H3K4me3 modified nucleosomes at the promoters of pluripotency regulators in the ground state [21] could recruit a redundant set of activating histone readers, masking the Ino80 dependency of *Oct4* expression.

To characterize functions for Ino80 during early embryonic development we performed RNA ISH of several differentiation markers in peri- and post-implantation embryos. Our analysis documents that the DVE markers *Cer1*, *Hex,* and *Lefty1* are not expressed in *Ino80* KO E5.5 embryos. DVE establishment involves several Smad signaling pathways including those activated by the Nodal and Bmp4 ligands [15]. Nodal was the first discovered ligand to promote the DVE [55], then later it was discovered that Bmp4 signaling repressed the DVE [15]. *Bmp4* is normally expressed in the ExE, which is proximally located from the distal tip of the embryo, and future site of the DVE [34]. During egg cylinder expansion (E4.5-E5.5), the ExE is rapidly moved away from the distal tip of the embryo. During this period, the repressive effects of Bmp4 on the distal tip of the embryo subside due to the increased distance between the ExE and distal tip during egg cylinder expansion [34], and activating Nodal signals become dominant due to increased *Nodal* expression in the EmE [55, 56]. In this model, the combined loss of Bmp4 signaling and increased Nodal signaling to the distal VE, establishes the DVE [15]. From our studies, we observed that *Nodal* is Ino80-independent during embryoid body differentiation and embryonic development. In contrast to *Nodal,* we observe that *Bmp4* is upregulated during embryoid body differentiation and in the E5.5 EmE. The abnormal expression of *Bmp4* in the EmE could result in down regulation of DVE markers [15]. Alternatively, defects in the DVE could result from general defects in the VE as supported by reduced *Hnf4a* expression with *Ino80* KO, or due to reduction in epiblast proliferation. Ino80 repression of *Bmp4* expression in the EmE could be direct because it localizes to, regulates chromatin structure and transcription factor occupancy at, the *Bmp4* promoter during ESC differentiation. In addition to these factors, we observed reduced *Bmp4* expression in the ExE, suggesting that Ino80 could also be an activator of *Bmp4* in extraembryonic tissues. These activities could also be direct, and the study of *Bmp4* expression in differentiating *Ino80* KO trophoblast stem cells could test this hypothesis [57].

## Conclusions

From the data presented in this manuscript, we show that Ino80 is required to pattern the early embryo. Using several models of development, we showed that *Ino80* KO embryos upregulate *Bmp4* expression, and when measurable, we observed a coincident repression of the DVE markers *Cer1*, *Hex,* and *Lefty1*. Because *Bmp4* represses the DVE, we propose that the lack of a DVE in *Ino80* KO embryos is in part due to elevated *Bmp4* expression in the EmE. Ino80 functions in regulating *Bmp4* expression are likely direct because we observed specific Ino80 localization to its promoter in differentiating ESCs. We also observed evidence that Ino80 remodels chromatin and regulates SP1 binding at the *Bmp4* promoter under these same conditions. These results support a model where Ino80 directly represses *Bmp4* expression in the EmE, and its repression is essential for DVE establishment and ultimately a functional P-D axis (Fig. 5h).

## Methods

### Gene targeting and animal husbandry

The RPC121 mouse PAC library (MRC, UK, London) was screened using probe P2 internal to the *Ino80* gene. Probe hybridization was performed using 0.25 M sodium phosphate pH 7.2, 1 mM EDTA, 7 % SDS at 65 °C overnight. Blots were washed three times for 30 minutes with 0.25XSSC, 0.1 % SDS at 65 °C. Five positive P1 clones were identified, and recombineering was used to move the chr2: 119,464,212-119,447,795 (mm10 assembly) DNA sequence from clone 386-C4 into DNDF-7. Recombineering was subsequently used to insert an upstream loxp site and a downstream frt-Neo-frt-loxp selection cassette.

Linearized *Ino80* targeting vector was electroporated into R1 ESC (129 Sv X 129Sv-CP F1) on feeder layers. Neomycin resistant clones were isolated and screened for successful *Ino80* targeting using a BglI digest and probe P2, BglI digest and probe P3, and an EcoRV digest using probe P4. Primers used to amplify probes from genomic DNA are available in Additional file 6. Southern blotting was performed as described for probe hybridization. *Ino80* was successfully targeted in 1 of 456 neomycin resistant clones. Germ line transmission of *Ino80* Floxed-Neo ESCs from two founder chimera mice was confirmed by Southern blotting.

The *Ino80* Floxed-Neo mice were crossed to Cre deleter (Jackson labs Cat#006054) and Flp deleter strains (Jackson labs Cat#011065) to create *Ino80* Floxed and *Ino80* KO alleles, respectively. *Ino80* Floxed and *Ino80* KO mice were then backcrossed to C57BL/6 J for five generations and confirmed Cre and Flp negative by PCR genotyping. Once backcrossed founder lines were established, the Ino80 Floxed mice were cryopreserved at Jackson Laboratory (Jackson labs Cat#027920). To create *Ino80* doxycycline inducible KO MEFs, the *Ino80* Floxed mice were crossed to TetO-Cre (Jackson Labs Cat#006234) and subsequently rtTA (Jackson Labs Cat#006965). MEFs were generated from an *Ino80* F/F, TetO-Cre hemizygous, rTTA homozygous X *Ino80* F/F, and rTTA homozygous cross. Genotypes of mice were determined by PCR using the *Ino80* genotyping protocol or those provided by Jackson Labs for purchased alleles. The *Ino80* genotyping protocol includes primers P1, P2, and P3 in a multiplex PCR reaction using recombinant Taq polymerase with Termal Pol Buffer (NEB). PCR conditions were as follows: 94 °C for three minutes for one cycle, 94 °C for 30 sec, 54 °C for 60 sec, 72 °C for 45 sec repeated for 40 cycles. Primer sequences are available as Additional file 6.

The mice used in this study were housed in a specific pathogen-free facility at Virginia Commonwealth University, Richmond VA, USA. Mice were maintained on a 12-hour light/dark cycle, and provided a low fat diet and hypochlorinated water *ad labium* throughout the duration of the study. All experiments and animal procedures were approved by the Animal Care and Use Committee of Virginia Commonwealth University under protocol AD10000372 and its modifications.

### Cell line isolation and maintenance

To create *Ino80* KO ESCs, we harvested and maintained E2.5-day embryos in KSOM media until the blastocyst stage. Expanded blastocysts were hatched using Tyrodes solution (Sigma T1788) and allowed to attach and outgrow on gelatinized plates for seven days in defined 2i + LIF media without serum (Millipore Cat# SF016-100). After outgrowth, the ICM was removed with a fine glass pipette, dispersed with 0.25 % trypsin + EDTA, and expanded in 2i + LIF + 15 % ESC grade serum (Life Technologies) media on gelatinized plates. Depending on the experiment, ESC lines were also grown in 2i + LIF growth media lacking serum (Millipore SF016-100), or a serum + LIF formulation (15 % ESC grade FCS (Life Technologies), DMEM, nonessential amino acids, 2 mM glutamine, 10 μM β-mercaptoethanol, penicillin, and streptomycin, 1000 U/ml LIF (Life Technologies)). ESC lines were genotyped by PCR methods (see above) and routinely maintained in serum + 2i + LIF containing media on gelatinized plates. *Ino80* KO at the protein level was confirmed by Western blotting using a custom rabbit polyclonal antibody and standard techniques. For gene expression studies, cells were harvested from culture using Tri-Reagent (Sigma) and total RNA purified according to the manufacture’s protocol. Quantitative RT-PCR was performed as described below.

Primary MEFs were produced from E12.5-day embryos using standard methods. MEFs were maintained in DMEM containing 10 % FCS, 2 mM glutamine, 1 mM nonessential amino acids, penicillin, and streptomycin. All experiments were performed using early passage (P0-1) fibroblasts. *Ino80* deletion was achieved by adding 10 ng/ml doxycycline to the growth media for two days. Following doxycycline exposure, the medium was replaced and MEFs were maintained at subconfluence using standard tissue culture techniques. Efficiency of *Ino80* deletion was confirmed by PCR as described above. Analysis of *Ino80* transcripts was accomplished by RT-PCR using Superscript II (Life Technologies) followed by amplification using Phusion polymerase (NEB). PCR fragments were cloned into pTOPO Blunt Zero vector (Life Technologies) and sequenced.

To stain for β-galactosidase activity, MEFs were washed in phosphate buffered saline (PBS) and then fixed in 4 % buffered formaldehyde for five minutes. Fixed cells were washed and then stained in 40 mM citric acid/sodium phosphate buffer, pH 6.0, containing 5 mM potassium ferrocyanide, 5 mM potassium ferricyanide, 150 mM sodium chloride, and 2 mM magnesium chloride overnight with gentle rocking. Cells were washed in PBS and blue cells counted with a microscope.

Annexin V + 7AAD staining of ESC lines was performed using AnnexinV-PE (BD Biosciences Cat#556421) using the protocol provided by BD Biosciences.

To stain for AP activity, ESC colonies were first briefly washed in PBS. Colonies were then fixed for five minutes at room temperature in PBS containing 0.2 % glutaraldehyde, 0.02 % NP-40, and 0.01 % sodium DOC. Colonies were then washed for ten minutes at room temperature in 100 mM Tris-HCl buffer, pH 9.5, containing 100 mM NaCl and 10 mM MgCl_2_. Colonies were then stained in BM purple AP substrate (Roche Cat# 11442074001) until the desired level of staining was achieved. Cells were then washed in PBS containing 0.1 % tween-20 and 2 mM MgCl_2_. The plates were then air dried.

To determine doubling time cells were plated at 5 × 10^5^ cells per well in a 24-well plate and incubated for the indicated time points, trypsinized, and counted using a hemocytometer. Doubling time was calculated using standard equations.

### Teratoma and embryoid body production

To produce teratomas, 1 × 10^6^ ESC were injected into the flank of NOD/SCID mice (Jackson Labs Cat# 001303), after which the tumors were allowed to grow for three weeks. The mice were then sacrificed, and the tumors were removed and frozen in liquid nitrogen. Tumors were sectioned in OCT medium using a cryostat, fixed in 10 % neutral buffered formalin, stained with hematoxylin and eosin, and visualized with light microscopy using standard techniques.

Embryoid bodies were created by transferring 10 × 10^6^ ESC into 13 × 100 mm polypropylene culture tubes with 3 ml DMEM containing 15 % ESC grade FCS (Life Technologies), nonessential amino acids, 2 mM glutamine, 10 μM β-mercaptoethanol, penicillin and streptomycin (2i + LIF withdrawal growth conditions). Upon harvest, embryoid bodies were dispersed using Tri-Reagent (Sigma) and total RNA was purified according to the manufacture’s protocol. Total RNA was then processed for quantitative RT-PCR as described below. Day 9 embryoid bodies were fixed in 10 % neutral buffered formalin overnight, washed in PBS, and then equilibrated in OCT before freezing and sectioning with a cryostat. Sections were stained with hematoxylin and eosin and imaged using light microscopy according to standard procedures.

### RNA in situ hybridization and TUNEL assays

Embryos were dissected from timed pregnancies and photographed or prepared for in situ hybridization as described previously [48]. Alkaline phosphatase (AP) coupled anti-DIG Fab fragments and BM Purple substrate (Roche) were used to detect DIG labeled antisense probes as previously described [48]. For co-localization staining, DIG-Bmp4 and FITC-Otx2 antisense labeled probes were detected by AP conjugated anti-DIG Fab and anti-FITC Fab fragment, subsequently. The two probes were visualized by two different AP substrates; the Bmp4 detection used BM Purple (Roche) and Otx2 detection used INT/BCIP (Roche) after inactivating the first AP activity (Otx2) with 4 % formaldehyde for 30 min.

To measure apoptosis E6.5 embryos were fixed for two hours at 4C, washed with PBST and kept in 100 % a methanol until use. Embryos were treated with 5:1 methanol:30 % hydrogen peroxide for three hours. Then, they were washed in a methanol and treated with 20 ug/ul proteinase K for three minutes and fixed with 0.2 % glutaraldehyde in 4 % paraformaldehyde for 20 minutes at room temperature. Embryos were washed three times with PBST and treated with sodium borohydride for 20 minutes. Embryos were then washed with TdT buffer and apoptotic cells were detected by TUNEL staining using a fluorescein in situ cell death detection kit (Sigma). Apoptosis was calculated for each embryo by averaging the percentage TUNEL+ cells (brown nuclei) to all nuclei (DAPI staining) in three representative 50 um fields using microscopy.

### Formaldehyde Assisted Isolation of Regulatory Elements (FAIRE)

FAIRE-qPCR was performed as described [58]. The qRT-PCR cycle threshold (*Ct)* value from the Input and FAIRE samples (control and *Ino80* KO) were normalized to signal from the reference region in each sample. Next, the relative enrichment for site in the *Bmp4* promoter in comparison to the reference primer set was calculated using the comparative ΔΔCt method. Primers used for FAIRE are available as Additional file 6.

### Chromatin immunoprecipitation (ChIP)

ChIP was performed as described previously [48]. Antibodies used include Ino80 (VCU23, custom rabbit polyclonal), SP1 (Santa Cruz Biotech # sc-59), pan histone H3 (Abcam # ab1791), and H3K4me3 (Abcam # ab1012). DNA quantification was accomplished using SsoAdvanced universal SYBR Green supermix (Biorad) according to the manufacturer’s established protocol. Data collection was performed using a 7900HT Fast Real Time PCR System (Applied Biosystems) and quantified as % Input DNA for Ino80 or SP1, or as a ratio of % Input H3K4me3/pan H3. The primers used for ChIP are available in Additional file 6.

Mouse Ino80 antibody VCU23 was raised in rabbits as a GST fusion to aa792-892 of Genbank # AAH59235.1. It was subsequently affinity purified by column chromatography using 6 × His-aa792-892. Specificity to Ino80 was confirmed by Western blotting using Ino80 control and KO ESC extracts. As a reference we observed identical results using the commercially available Ino80 antibody from ProteinTech (Cat# 18810-1-AP) (See Additional file 7).

### Gene expression assays

Quantitative RT-PCR was performed on cDNA libraries converted using Superscript II (Life Technologies). cDNA libraries were amplified using SsoAdvanced universal SYBR green supermix (Biorad) according to the manufacturer’s established protocols. Primers used for gene specific quantitative PCR were previously described [48]. Data collection was performed using a 7900HT Fast Real Time PCR System (Applied Biosystems) and the ΔΔCt method was used to estimate transcript abundance relative to the normalization control *Gapdh*.

Northern blotting was performed as previously described [48]. A total of 1 ug of polyA+ RNA was resolved by 1 % formaldehyde agarose electrophoresis and transferred to Hybond N+ (GE Health Sciences) using the manufacturer’s suggested protocol. The *Ino80* probe was amplified from ESC cDNA library using Taq polymerase, sequenced to confirm identity and labeled with P32 using random hexamer labeling. Detection was performed by phosphoimager.

### Statistics

Standard deviation was used to calculate error bars throughout the study. *P* values were calculated using two tailed T tests. Number of replicates are designated in the figure legend.

### Availability of supporting data

Data supporting the results of this article are available in Additional file 8.

## Additional files

Additional file 1: Figure S1. Targeting and genotyping strategy for conditional and knockout *Ino80* alleles. a Southern blotting strategy for screening embryonic stem cell clones successfully integrating the *Ino80* targeting vector. Probes used in the screen are positioned at the site of hybridization, are designated in red, and are labeled P2, P3, and P4. Restriction enzyme sites are designated above the genome sequence. Loxp and Neo features of the targeting vector are shown in black. Exons are shown as vertical lines and designated by number in sequential order from 5′ to 3′. b Results of Southern blotting experiments to confirm successful integration through Cre- or Flp-mediated recombination of the Ino80 targeting vector. Wild-type targeted *Ino80* Floxed-Neo (Neo), *Ino80* Floxed (Floxed) and *Ino80* knockout (KO) alleles are shown. DNA fragment sizes obtained from restriction enzyme digestion and Southern blotting with P2, P3, and P4 probes are as expected from diagrams in panel a. c Diagram describing PCR-based genotyping strategy. *Ino80* alleles as defined in panel a are shown in a cartoon format with exons and introns not to scale. Position of the Loxp sites (red dots) and the Neo selectable marker are shown relative to *Ino80* exons 2-4, which are designated by numbers. Positions of primers P1, P2, and P3 are shown as arrows designating 5′-3′ orientation. PCR amplicons and their size are shown as black bars above amplifying primers. d Results from the PCR genotyping strategy using DNA from wild type and mice heterozygous for the *Ino80* Floxed-Neo, *Ino80* Floxed, and *Ino80* KO alleles. (TIF 10353 kb) Additional file 2: Figure S2. Molecular characterization of the *Ino80* KO allele. a Efficiency of Cre-mediated excision of *Ino80* exons 2-4 from control *Ino80* Floxed/Floxed (Cre-Neg) and *Ino80* Floxed/Floxed, TetO-Cre (TetO-Cre) primary MEFs were monitored using the PCR genotyping strategy. Cells were incubated with 10 μg/ml doxycycline for two days, and then shifted to culture medium without doxycycline. DNA was taken for analysis prior to the addition of doxycycline (Day 0), and at two, four and ten days post-doxycycline addition. b Western blot analysis of Ino80 protein from total cell extracts prior to doxycycline addition (Day 0) and six days after initial doxycycline treatment using a custom Ino80 antibody. Ponceau S was used as a loading control. c Cartoon showing the position of RT-PCR primers used for characterizing the *Ino80* KO allele transcript. Location of the forward primer in exon 1 and reverse primer in exon 7 are shown. The position of the loxp sites flanking exons 2-4 are shown as black boxes. The position of the initiating ATG is shown in exon 2. d RT-PCR results using primers shown in panel c on cDNAs converted from total RNA harvested from control *Ino80* Floxed/Floxed (Cre-Neg) and *Ino80* Floxed/Floxed, TetO-Cre (TetO-Cre) MEFs six days post-doxycycline treatment. PCR products were gel purified, cloned and sequenced. Exon composition of PCR fragments is shown. e Predicted reading frames for the KO-1 and KO-2 transcripts initiating from the first available ATG. (TIF 10038 kb) Additional file 3: Genotyping results from intercross of heterozygous *Ino80* KO mice. Litters of mice or embryos at specified stages were collected and genotyped using the PCR-based genotyping strategy. Significant deviations from the expected genotype ratios were identified using a Chi-Square test (***p* < 0.01). (XLSX 10 kb) Additional file 4: Figure S4. Ino80 is not essential for the growth and viability of ESCs maintained in ground state pluripotency. a Growth curve of wild-type and *Ino80* KO ESCs. 1.0 × 10^4^ cells were seeded onto gelatinized plates containing serum + 2i + LIF media. Numbers of trypan blue negative cells were counted every day over a four-day period. (Doubling Time, WT = 36 ± 4.5 hours, KO = 44 ± 4.2 hours; t test p ≤0.05, N = 3 biological replicates) b AnnexinV + 7AAD staining of wild-type and *Ino80* KO ESCs maintained in serum + 2i + LIF (*N* = 3 biological replicates). (TIF 8494 kb) Additional file 5: Figure S5.
*Ino80* is widely expressed in adult tissues. Abundance of *Ino80* transcripts in a variety of adult tissues was determined by Northern blotting. Ethidium bromide stained rRNA serves as a loading control. (TIF 1828 kb) Additional file 6: Primers used in this Study. (XLSX 12 kb) Additional file 7: Figure S7. Western blotting confirming custom Ino80 antibody. Western analysis of Ino80 protein expression in wild-type and Ino80 KO ESCs using custom antibody VCU23 (a) and a commercially available Ino80 antibody (ProteinTech Cat# 18810-1-AP) (b). In each case Ponceau S was used as a loading control. (TIF 6874 kb) Additional file 8: Supporting data. (XLSX 36 kb)

## References

[1] Tam PP, Loebel DA (2007). Gene function in mouse embryogenesis: get set for gastrulation. Nat Rev Genet.

[2] Kojima Y, Tam OH, Tam PP (2014). Timing of developmental events in the early mouse embryo. Semin Cell Dev Biol.

[3] Cutter AR, Hayes JJ (2015). A brief review of nucleosome structure. FEBS Lett.

[4] Li G, Zhu P (2015). Structure and organization of chromatin fiber in the nucleus. FEBS Lett.

[5] Becker PB, Workman JL (2013). Nucleosome remodeling and epigenetics. Cold Spring Harb Perspect Biol.

[6] Gerhold CB, Gasser SM (2014). INO80 and SWR complexes: relating structure to function in chromatin remodeling. Trends Cell Biol.

[7] Fritsch O, Benvenuto G, Bowler C, Molinier J, Hohn B (2004). The INO80 protein controls homologous recombination in Arabidopsis thaliana. Mol Cell.

[8] Zhang C, Cao L, Rong L, An Z, Zhou W, Ma J (2015). The chromatin-remodeling factor AtINO80 plays crucial roles in genome stability maintenance and in plant development. Plant J.

[9] Bhatia S, Pawar H, Dasari V, Mishra RK, Chandrashekaran S, Brahmachari V (2010). Chromatin remodeling protein INO80 has a role in regulation of homeotic gene expression in Drosophila. Genes Cells.

[10] Neuman SD, Ihry RJ, Gruetzmacher KM, Bashirullah A (2014). INO80-dependent regression of ecdysone-induced transcriptional responses regulates developmental timing in Drosophila. Dev Biol.

[11] Moshkin YM, Chalkley GE, Kan TW, Reddy BA, Ozgur Z, van Ijcken WF (2012). Remodelers organize cellular chromatin by counteracting intrinsic histone-DNA sequence preferences in a class-specific manner. Mol Cell Biol.

[12] Lee HS, Lee SA, Hur SK, Seo JW, Kwon J (2014). Stabilization and targeting of INO80 to replication forks by BAP1 during normal DNA synthesis. Nat Commun.

[13] Wang L, Du Y, Ward JM, Shimbo T, Lackford B, Zheng X (2014). INO80 facilitates pluripotency gene activation in embryonic stem cell self-renewal, reprogramming, and blastocyst development. Cell Stem Cell.

[14] Min JN, Tian Y, Xiao Y, Wu L, Li L, Chang S (2013). The mINO80 chromatin remodeling complex is required for efficient telomere replication and maintenance of genome stability. Cell Res.

[15] Yamamoto M, Beppu H, Takaoka K, Meno C, Li E, Miyazono K (2009). Antagonism between Smad1 and Smad2 signaling determines the site of distal visceral endoderm formation in the mouse embryo. J Cell Biol.

[16] Bouabe H, Okkenhaug K (2013). Gene targeting in mice: a review. Methods Mol Biol.

[17] Hochedlinger K, Yamada Y, Beard C, Jaenisch R (2005). Ectopic expression of Oct-4 blocks progenitor-cell differentiation and causes dysplasia in epithelial tissues. Cell.

[18] Perl AK, Wert SE, Nagy A, Lobe CG, Whitsett JA (2002). Early restriction of peripheral and proximal cell lineages during formation of the lung. Proc Natl Acad Sci U S A.

[19] Dimri GP, Lee X, Basile G, Acosta M, Scott G, Roskelley C (1995). A biomarker that identifies senescent human cells in culture and in aging skin in vivo. Proc Natl Acad Sci U S A.

[20] Ying QL, Wray J, Nichols J, Batlle-Morera L, Doble B, Woodgett J (2008). The ground state of embryonic stem cell self-renewal. Nature.

[21] Marks H, Kalkan T, Menafra R, Denissov S, Jones K, Hofemeister H (2012). The transcriptional and epigenomic foundations of ground state pluripotency. Cell.

[22] Hultman I, Bjork L, Blomberg E, Sandstedt B, Ahrlund-Richter L (2014). Experimental teratoma: at the crossroad of fetal- and onco-development. Semin Cancer Biol.

[23] Weitzer G (2006). Embryonic stem cell-derived embryoid bodies: an in vitro model of eutherian pregastrulation development and early gastrulation. Handb Exp Pharmacol.

[24] Coucouvanis E, Martin GR (1999). BMP signaling plays a role in visceral endoderm differentiation and cavitation in the early mouse embryo. Development.

[25] Cai KQ, Capo-Chichi CD, Rula ME, Yang DH, Xu XX (2008). Dynamic GATA6 expression in primitive endoderm formation and maturation in early mouse embryogenesis. Dev Dyn.

[26] Ying QL, Nichols J, Chambers I, Smith A (2003). BMP induction of Id proteins suppresses differentiation and sustains embryonic stem cell self-renewal in collaboration with STAT3. Cell.

[27] Nemer G, Nemer M (2003). Transcriptional activation of BMP-4 and regulation of mammalian organogenesis by GATA-4 and -6. Dev Biol.

[28] Hebert JM, Basilico C, Goldfarb M, Haub O, Martin GR (1990). Isolation of cDNAs encoding four mouse FGF family members and characterization of their expression patterns during embryogenesis. Dev Biol.

[29] Duncan SA, Manova K, Chen WS, Hoodless P, Weinstein DC, Bachvarova RF (1994). Expression of transcription factor HNF-4 in the extraembryonic endoderm, gut, and nephrogenic tissue of the developing mouse embryo: HNF-4 is a marker for primary endoderm in the implanting blastocyst. Proc Natl Acad Sci U S A.

[30] Crossley PH, Martin GR (1995). The mouse Fgf8 gene encodes a family of polypeptides and is expressed in regions that direct outgrowth and patterning in the developing embryo. Development.

[31] Wilkinson DG, Bhatt S, Herrmann BG (1990). Expression pattern of the mouse T gene and its role in mesoderm formation. Nature.

[32] Stower MJ, Srinivas S (2014). Heading forwards: anterior visceral endoderm migration in patterning the mouse embryo. Philos Trans R Soc Lond B Biol Sci.

[33] Richardson L, Torres-Padilla ME, Zernicka-Goetz M (2006). Regionalised signalling within the extraembryonic ectoderm regulates anterior visceral endoderm positioning in the mouse embryo. Mech Dev.

[34] Winnier G, Blessing M, Labosky PA, Hogan BL (1995). Bone morphogenetic protein-4 is required for mesoderm formation and patterning in the mouse. Genes Dev.

[35] Ang SL, Conlon RA, Jin O, Rossant J (1994). Positive and negative signals from mesoderm regulate the expression of mouse Otx2 in ectoderm explants. Development.

[36] Robertson EJ (2014). Dose-dependent Nodal/Smad signals pattern the early mouse embryo. Semin Cell Dev Biol.

[37] Mesnard D, Guzman-Ayala M, Constam DB (2006). Nodal specifies embryonic visceral endoderm and sustains pluripotent cells in the epiblast before overt axial patterning. Development.

[38] Stephenson RO, Rossant J, Tam PP. Intercellular interactions, position, and polarity in establishing blastocyst cell lineages and embryonic axes. Cold Spring Harb Perspect Biol 2012, 4(11): a008235. doi:10.1101/cshperspect.a008235. 10.1101/cshperspect.a008235 PMC3536338 23125013

[39] Sharova LV, Sharov AA, Piao Y, Shaik N, Sullivan T, Stewart CL (2007). Global gene expression profiling reveals similarities and differences among mouse pluripotent stem cells of different origins and strains. Dev Biol.

[40] Zhu W, Yao X, Liang Y, Liang D, Song L, Jing N (2015). Mediator Med23 deficiency enhances neural differentiation of murine embryonic stem cells through modulating BMP signaling. Development.

[41] Harikumar A, Meshorer E (2015). Chromatin remodeling and bivalent histone modifications in embryonic stem cells. EMBO Rep.

[42] Ebara S, Kawasaki S, Nakamura I, Tsutsumimoto T, Nakayama K, Nikaido T (1997). Transcriptional regulation of the mBMP-4 gene through an E-box in the 5′-flanking promoter region involving USF. Biochem Biophys Res Commun.

[43] Ho L, Crabtree GR (2010). Chromatin remodelling during development. Nature.

[44] Chen T, Dent SY (2014). Chromatin modifiers and remodellers: regulators of cellular differentiation. Nat Rev Genet.

[45] Bultman S, Gebuhr T, Yee D, La Mantia C, Nicholson J, Gilliam A (2000). A Brg1 null mutation in the mouse reveals functional differences among mammalian SWI/SNF complexes. Mol Cell.

[46] Kaji K, Nichols J, Hendrich B (2007). Mbd3, a component of the NuRD co-repressor complex, is required for development of pluripotent cells. Development.

[47] Thomas T, Dixon MP, Kueh AJ, Voss AK (2008). Mof (MYST1 or KAT8) is essential for progression of embryonic development past the blastocyst stage and required for normal chromatin architecture. Mol Cell Biol.

[48] Landry J, Sharov AA, Piao Y, Sharova LV, Xiao H, Southon E (2008). Essential role of chromatin remodeling protein Bptf in early mouse embryos and embryonic stem cells. PLoS Genet.

[49] Hurd EA, Capers PL, Blauwkamp MN, Adams ME, Raphael Y, Poucher HK (2007). Loss of Chd7 function in gene-trapped reporter mice is embryonic lethal and associated with severe defects in multiple developing tissues. Mamm Genome.

[50] Batista LF (2014). Telomere biology in stem cells and reprogramming. Prog Mol Biol Transl Sci.

[51] Momcilovic O, Navara C, Schatten G (2011). Cell cycle adaptations and maintenance of genomic integrity in embryonic stem cells and induced pluripotent stem cells. Results Probl Cell Differ.

[52] Rais Y, Zviran A, Geula S, Gafni O, Chomsky E, Viukov S (2013). Deterministic direct reprogramming of somatic cells to pluripotency. Nature.

[53] Lanner F, Lee KL, Sohl M, Holmborn K, Yang H, Wilbertz J (2010). Heparan sulfation-dependent fibroblast growth factor signaling maintains embryonic stem cells primed for differentiation in a heterogeneous state. Stem Cells.

[54] Silva J, Nichols J, Theunissen TW, Guo G, van Oosten AL, Barrandon O (2009). Nanog is the gateway to the pluripotent ground state. Cell.

[55] Brennan J, Lu CC, Norris DP, Rodriguez TA, Beddington RS, Robertson EJ (2001). Nodal signalling in the epiblast patterns the early mouse embryo. Nature.

[56] Kumar A, Lualdi M, Lyozin GT, Sharma P, Loncarek J, Fu XY (2015). Nodal signaling from the visceral endoderm is required to maintain Nodal gene expression in the epiblast and drive DVE/AVE migration. Dev Biol.

[57] Roberts RM, Fisher SJ (2011). Trophoblast stem cells. Biol Reprod.

[58] Giresi PG, Lieb JD (2009). Isolation of active regulatory elements from eukaryotic chromatin using FAIRE (Formaldehyde Assisted Isolation of Regulatory Elements). Methods.

