# Supplementary figures and images for: Ino80 is essential for proximal-distal axis asymmetry in part by regulating Bmp4 expression
Source: BMC Biol. 2016 Mar 14;14:18. doi: 10.1186/s12915-016-0238-5 (PMC4790052; doi:10.1186/s12915-016-0238-5)

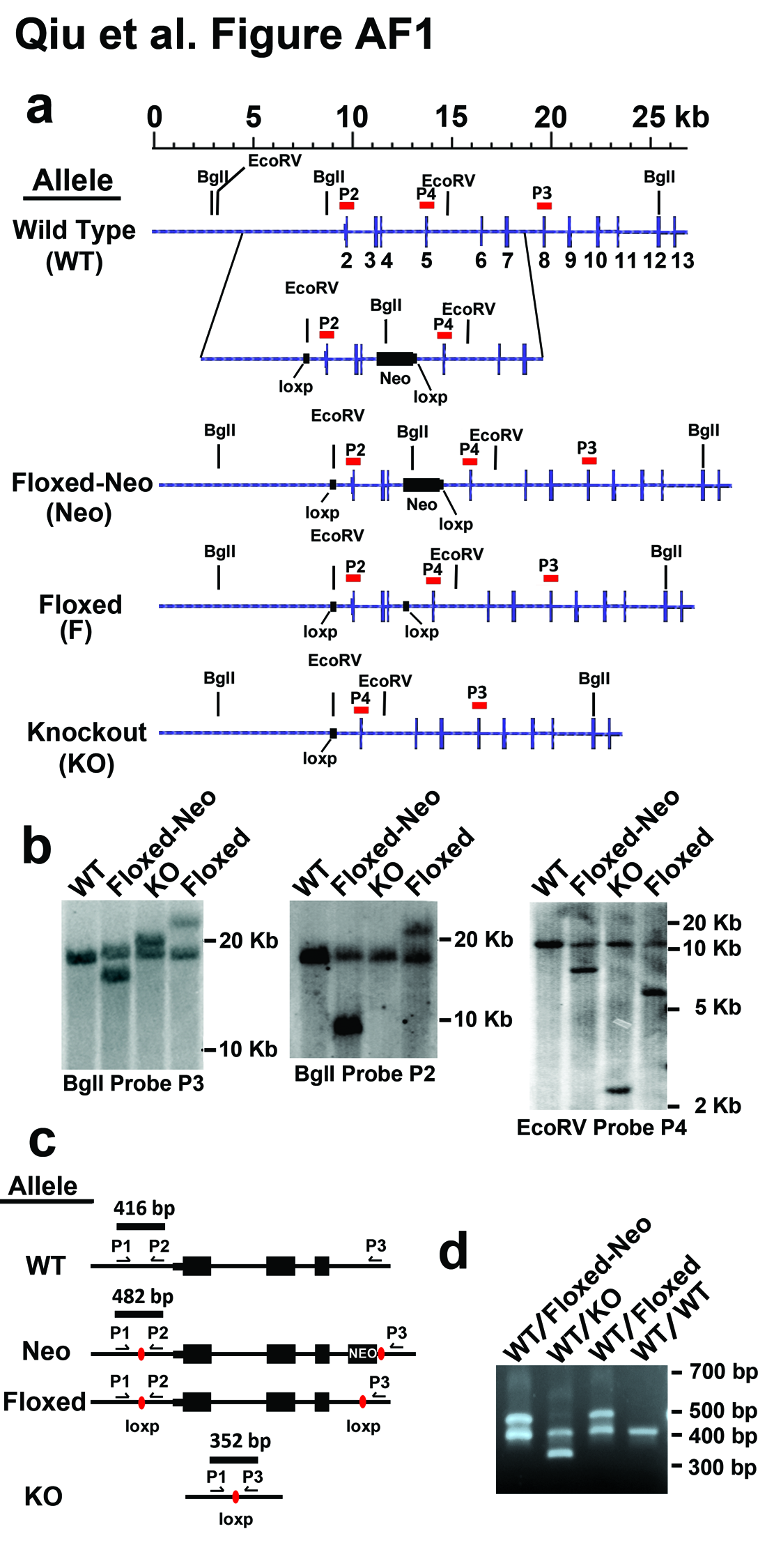

Supplement: Additional file 1: Figure S1. — Targeting and genotyping strategy for conditional and knockout Ino80 alleles. a Southern blotting strategy for screening embryonic stem cell clones successfully integrating the Ino80 targeting vector. Probes used in the screen are positioned at the site of hybridization, are designated in red, and are labeled P2, P3, and P4. Restriction enzyme sites are designated above the genome sequence. Loxp and Neo features of the targeting vector are shown in black. Exons are shown as vertical lines and designated by number in sequential order from 5′ to 3′. b Results of Southern blotting experiments to confirm successful integration through Cre- or Flp-mediated recombination of the Ino80 targeting vector. Wild-type targeted Ino80 Floxed-Neo (Neo), Ino80 Floxed (Floxed) and Ino80 knockout (KO) alleles are shown. DNA fragment sizes obtained from restriction enzyme digestion and Southern blotting with P2, P3, and P4 probes are as expected from diagrams in panel a. c Diagram describing PCR-based genotyping strategy. Ino80 alleles as defined in panel a are shown in a cartoon format with exons and introns not to scale. Position of the Loxp sites (red dots) and the Neo selectable marker are shown relative to Ino80 exons 2-4, which are designated by numbers. Positions of primers P1, P2, and P3 are shown as arrows designating 5′-3′ orientation. PCR amplicons and their size are shown as black bars above amplifying primers. d Results from the PCR genotyping strategy using DNA from wild type and mice heterozygous for the Ino80 Floxed-Neo, Ino80 Floxed, and Ino80 KO alleles. (TIF 10353 kb) [file 12915_2016_238_MOESM1_ESM.tif]

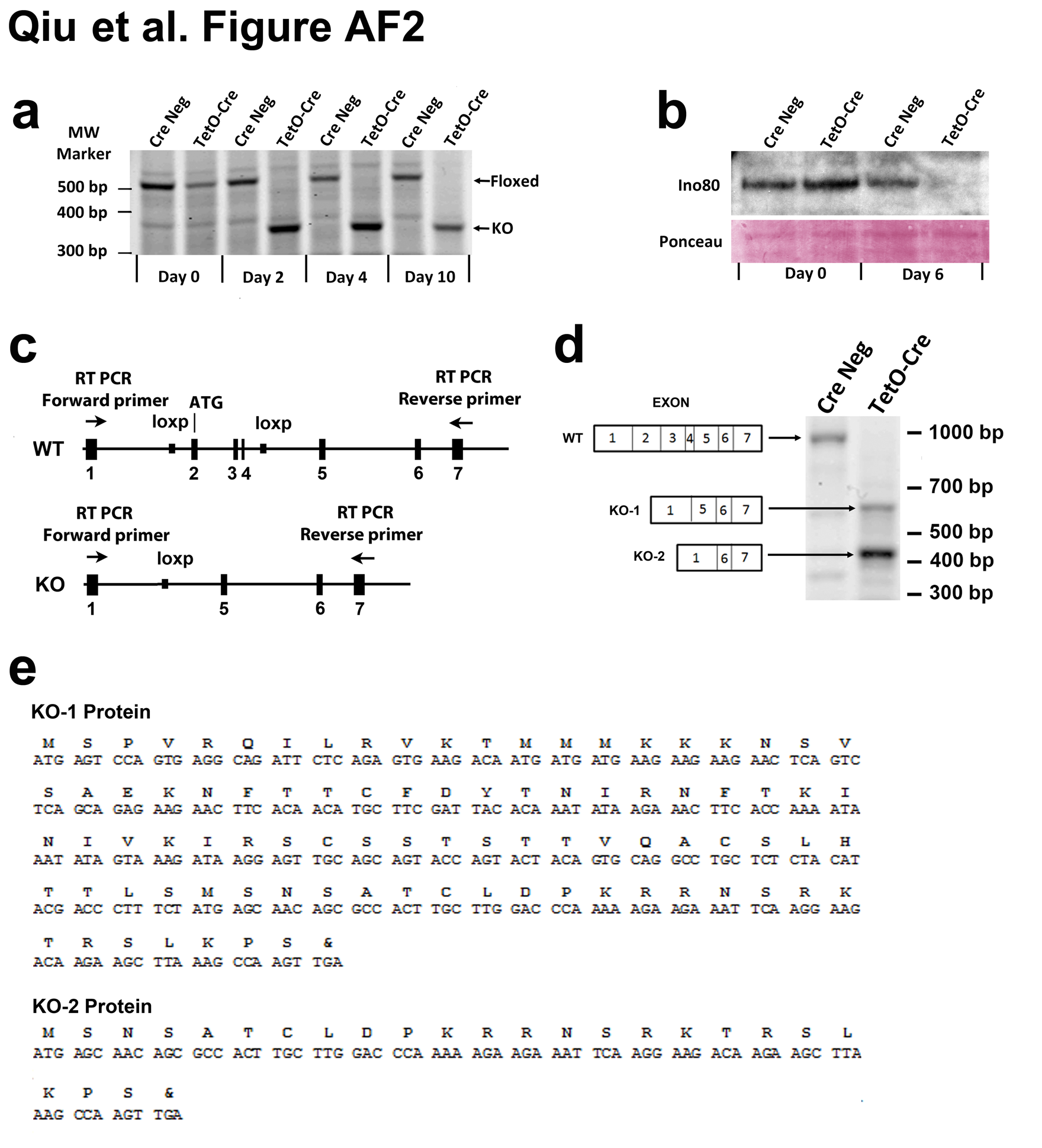

Supplement: Additional file 2: Figure S2. — Molecular characterization of the Ino80 KO allele. a Efficiency of Cre-mediated excision of Ino80 exons 2-4 from control Ino80 Floxed/Floxed (Cre-Neg) and Ino80 Floxed/Floxed, TetO-Cre (TetO-Cre) primary MEFs were monitored using the PCR genotyping strategy. Cells were incubated with 10 μg/ml doxycycline for two days, and then shifted to culture medium without doxycycline. DNA was taken for analysis prior to the addition of doxycycline (Day 0), and at two, four and ten days post-doxycycline addition. b Western blot analysis of Ino80 protein from total cell extracts prior to doxycycline addition (Day 0) and six days after initial doxycycline treatment using a custom Ino80 antibody. Ponceau S was used as a loading control. c Cartoon showing the position of RT-PCR primers used for characterizing the Ino80 KO allele transcript. Location of the forward primer in exon 1 and reverse primer in exon 7 are shown. The position of the loxp sites flanking exons 2-4 are shown as black boxes. The position of the initiating ATG is shown in exon 2. d RT-PCR results using primers shown in panel c on cDNAs converted from total RNA harvested from control Ino80 Floxed/Floxed (Cre-Neg) and Ino80 Floxed/Floxed, TetO-Cre (TetO-Cre) MEFs six days post-doxycycline treatment. PCR products were gel purified, cloned and sequenced. Exon composition of PCR fragments is shown. e Predicted reading frames for the KO-1 and KO-2 transcripts initiating from the first available ATG. (TIF 10038 kb) [file 12915_2016_238_MOESM2_ESM.tif]

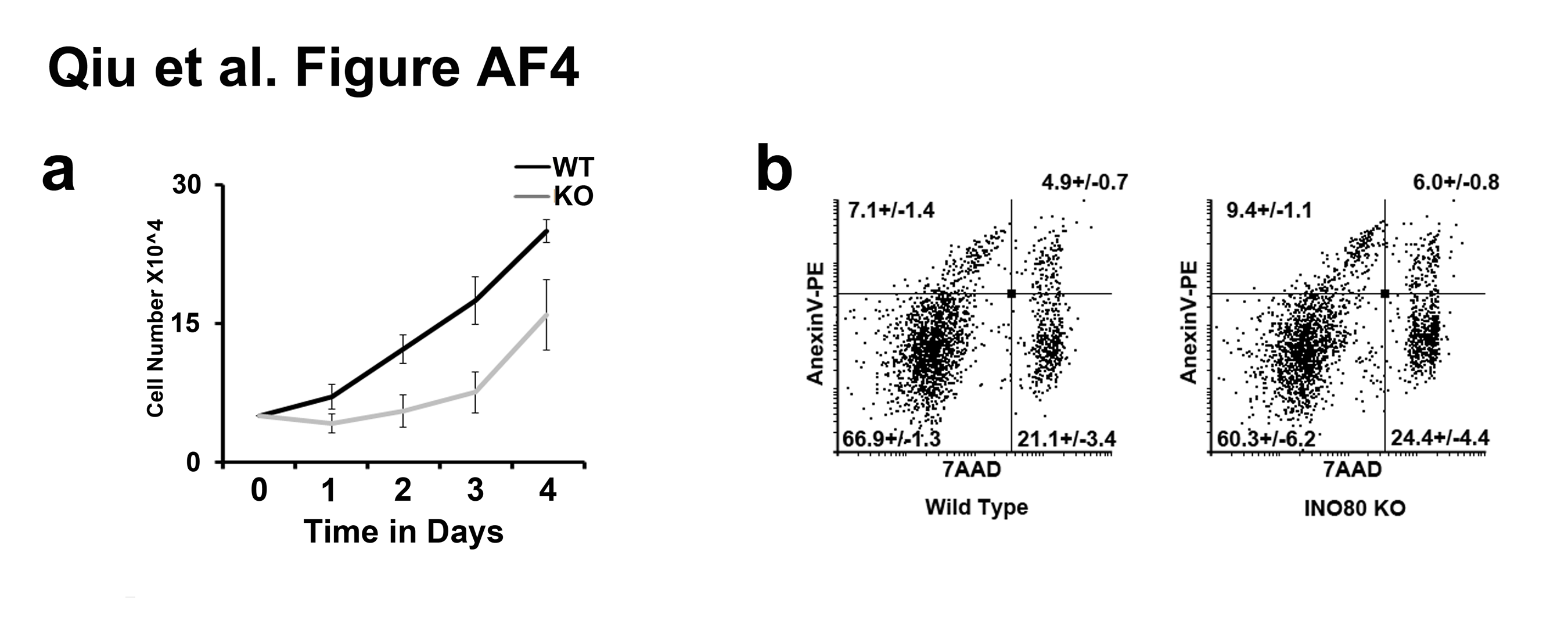

Supplement: Additional file 4: Figure S4. — Ino80 is not essential for the growth and viability of ESCs maintained in ground state pluripotency. a Growth curve of wild-type and Ino80 KO ESCs. 1.0 × 10^4^ cells were seeded onto gelatinized plates containing serum + 2i + LIF media. Numbers of trypan blue negative cells were counted every day over a four-day period. (Doubling Time, WT = 36 ± 4.5 hours, KO = 44 ± 4.2 hours; t test p ≤0.05, N = 3 biological replicates) b AnnexinV + 7AAD staining of wild-type and Ino80 KO ESCs maintained in serum + 2i + LIF (N = 3 biological replicates). (TIF 8494 kb) [file 12915_2016_238_MOESM4_ESM.tif]

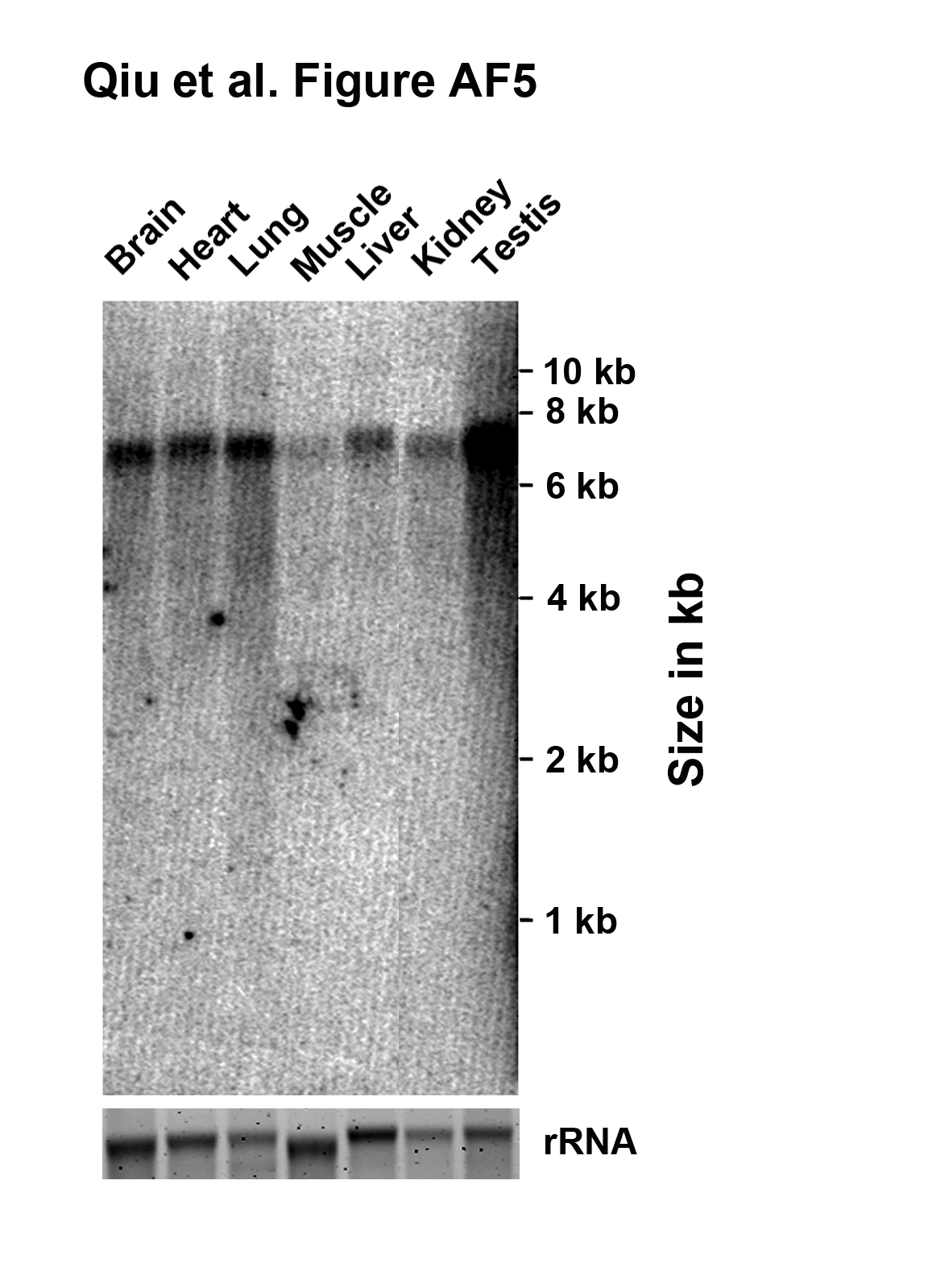

Supplement: Additional file 5: Figure S5. — Ino80 is widely expressed in adult tissues. Abundance of Ino80 transcripts in a variety of adult tissues was determined by Northern blotting. Ethidium bromide stained rRNA serves as a loading control. (TIF 1828 kb) [file 12915_2016_238_MOESM5_ESM.tif]

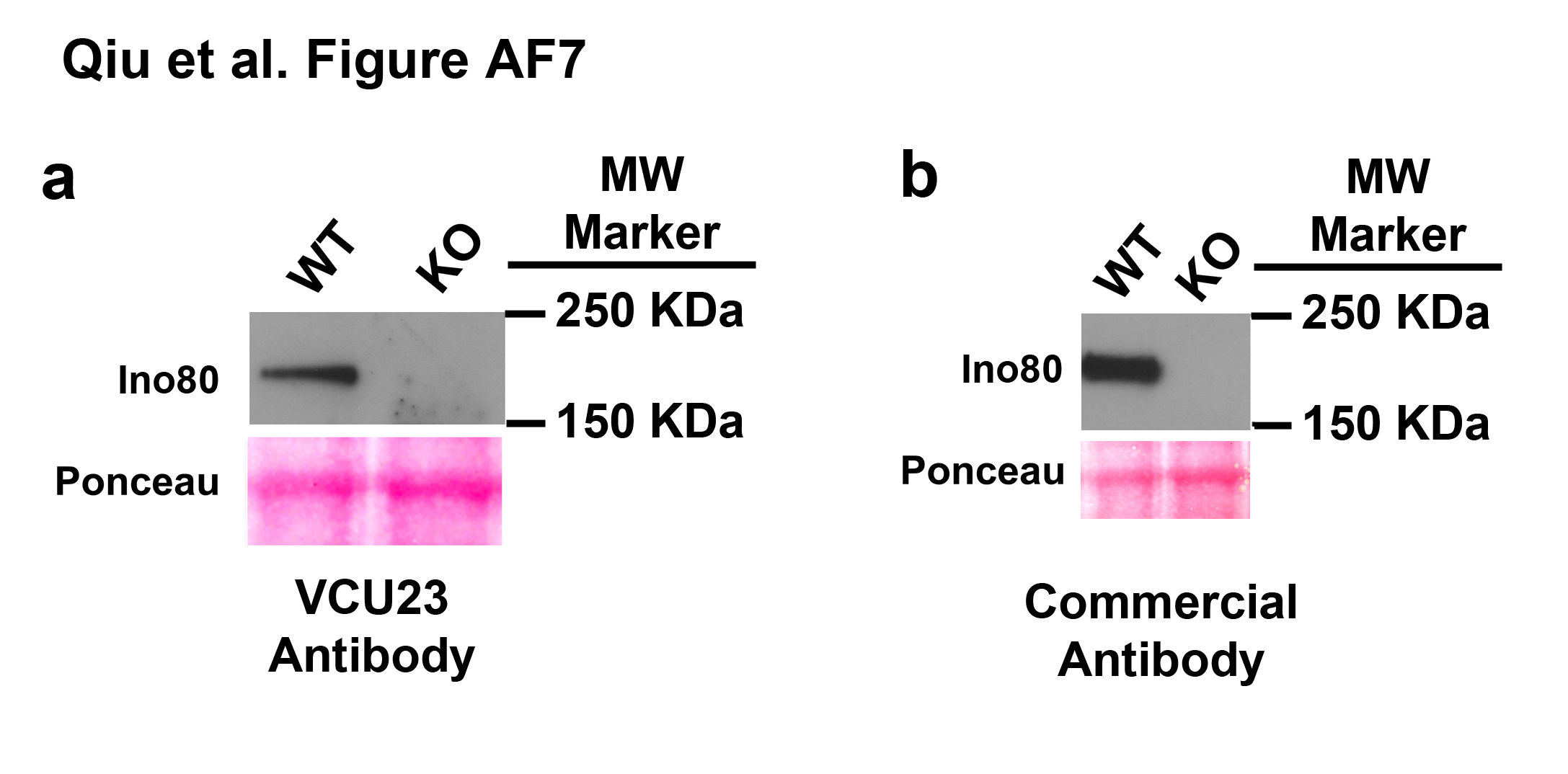

Supplement: Additional file 7: Figure S7. — Western blotting confirming custom Ino80 antibody. Western analysis of Ino80 protein expression in wild-type and Ino80 KO ESCs using custom antibody VCU23 (a) and a commercially available Ino80 antibody (ProteinTech Cat# 18810-1-AP) (b). In each case Ponceau S was used as a loading control. (TIF 6874 kb) [file 12915_2016_238_MOESM7_ESM.tif]
